# Editorial: Inflammation and Myeloid Cells in Cancer Progression and Metastasis

**DOI:** 10.3389/fcell.2022.913595

**Published:** 2022-04-29

**Authors:** Sanjay Mishra, Dinesh K. Ahirwar, Amit Kumar Srivastava, Prem Prakash Tripathi, Ramesh K. Ganju

**Affiliations:** ^1^ Department of Pathology, College of Medicine, The Ohio State University, Columbus, OH, United States; ^2^ Comprehensive Cancer Center, The Ohio State University, Columbus, OH, United States; ^3^ Cancer Biology & Inflammatory Disorder Division, CSIR-Indian Institute of Chemical Biology, Kolkata, India; ^4^ Cell Biology & Physiology, CSIR-Indian Institute of Chemical Biology, Kolkata, India

**Keywords:** cancer, inflammation, myeloid-derived suppressor cell, tumor micoenvironment, immunothearpy

Chronic inflammation predisposes the development, progression, and metastasis of cancer. Mutual interactions between tumor and inflammatory immune cells, especially myeloid cells, develop an inflammatory tumor microenvironment (TME) (Ahirwar et al., 2021; Mishra et al., 2022). Despite significant advances made over the past few decades, our knowledge of the molecular basis of inflammation-induced tumorigenesis and its role in regulating the myeloid cell’s functions remains very limited to a certain type of malignancies. Therefore, an in-depth understanding of inflammation-mediated interactions between tumors and myeloid cells may provide novel insights into the cellular and molecular mechanisms contributing to drug resistivity and metastatic traits. Moreover, it will help identify specific inflammation-associated genes which could be used as promising prognostic biomarkers and therapeutic targets to overcome the acquired resistivity to different types of existing chemo or immunotherapies.

Currently, limited information is available on the modulation of the inflammatory tumor immune microenvironment (TIME) that can address the higher incidence and aggressiveness of certain types of human cancers. The following articles on this research topic provide a comprehensive understanding of the different molecular mechanisms associated with the onset, progression, and metastasis of different human cancers with a particular interest in the role of myeloid cells. In this special research theme, we have received three research articles that entail the role of different genes associated with inflammation, immune modulation, and cancer progression. In addition, we accepted four review articles that highlight the functional role of inflammation and myeloid cells in enhancing tumorigenesis.

The immunosuppressive myeloid cells such as M2-tumor-associated macrophages (TAMs) and immature polymorphonuclear leukocytes (PMNs) have been shown to generate an immunosuppressive TME that initiate tumor development and enhance cancer progression and metastasis. M2-TAMs and PMN are known to induce immune suppression by inhibiting T cells (Peranzoni et al., 2018; Singel et al., 2019). In this research theme, Cheng et al. reviewed the functional role of myeloid-derived suppressor cells (MDSCs) in negatively regulating immune response, enhancing tumor progression and undermining immunotherapy efficiency. The authors described multiple MDSCs-mediated complex molecular mechanisms that modulate tumor growth and metastasis. This includes the generation of immunosuppressive TME which limits the anti-tumor T cell’s function and several non-immune roles such as enhancing the cancer stem-like properties and promoting angiogenesis. Furthermore, they discussed the negative impact of MDSCs on therapeutic responses and how they could be used as a prognostic marker for different human malignancies. Finally, the authors reviewed the therapeutic benefits of targeting MDSCs in suppressing the immunosuppressive TME, thereby enhancing the therapeutic response of chemotherapy in combination with immunotherapy. In another review article, Li et al., reviewed the literature on the role of tumor-infiltrating myeloid cells in limiting the host anti-tumor immunity that leads to the failure of many cancer immunotherapies. The authors elaborated that how molecular mechanisms which regulate the activity of the myeloid cells within the TME are targeted and that may overcome immunotherapy resistance in a few types of cancers. In this review, the authors discussed the importance of immunometabolism in modulating phenotypic plasticity as well as the myeloid cell’s functions. They revealed how the metabolism of intratumoral myeloid cells is rewired, which helps them adapt to the nutrition-deprived TME, thereby enhancing their pro-tumor phenotypes. Moreover, they discussed the metabolic shift that converts these immunosuppressive myeloid cells toward the anti-tumor immune-stimulating phenotype.

Additionally, Deng and Fleming research group described the functional role of inflammation in regulating the myeloid cells associated with cancer progression and metastasis. In this review, the authors described the role of different extrinsic and intrinsic factors that affect the host TME and regulate the plasticity and differentiation of myeloid cells. The authors reviewed the functional role of different activating mutations and TME milieu that regulate the anti or pro-inflammatory signaling cascade required for the differentiation of myeloid cells. Finally, they made comments on the advancement of novel immunotherapeutic strategies regarding myeloid-associated innate immunity.

Notably, there is growing evidence that inflammation with increased infiltration of myeloid cells plays a crucial role in cancer progression and metastasis which Deng and Fleming, also discussed in this special research theme. Another interesting review article by Begum et al. revealed how different types of metalloproteinases (MMPs) present in the TME play an important role in regulating inflammation-mediated gynecological malignancies. In this review, the authors highlight the functional role of MMPs in regulating gynecological cancer progression and immune surveillance evasion. This review discussed the contribution of different MMPs in regulating gynecological diseases and their inhibition for therapeutic intervention.

Chronic inflammation is also known to regulate the expression of several genes associated with altered molecular pathways and immune signatures in different solid tumors including breast cancer (Suman et al., 2016). In our research topic, the article published by Li et al. reported a comprehensive study of regulatory factors and immune-associated patterns to decode common BRCA1/2 mutation-type-specific crucial regulations in breast cancer. Their research studies found that BRCA1/2 mutations are associated with increased infiltration of immune cells such as follicular helper T cells, regulatory T cells, helper T cells (Th1 cells) as well as the exhaustion of T cells. Finally, Karmakar et al. demonstrated the aberrant expression of three genes (E2F3, ESR1, and UNC5D) in Retinoblastoma (Rb) tumors using different computational approaches and *in vitro* experiments. Altogether, their study demonstrated that E2F3, ESR1, and UNC5D may be fundamentally implicated in Rb tumorigenesis and could be used as promising early diagnostic biomarkers and therapeutic targets of Rb.

Another interesting research article published by Talebian et al., demonstrated the functional role of the CD200-CD200R signaling pathway in regulating the immune responses in melanoma. The authors first examined the growth of Yumm1.7 melanoma tumors using CD200R^−/-^ or anti-CD200 treated preclinical mouse models. They discovered that genetic or pharmacological inhibition of CD200 enhances the growth of melanoma cells through decreased infiltration of immune cells and TCR clonality. Moreover, the authors also observed the decreased numbers of IFN-γ^+^ and TNF-α^+^ T cells in these preclinical mouse models. Furthermore, the authors discuss the upregulation of macrophage-derived CCL8 in the absence of CD200-CD200R interaction. In summary, this Frontiers special topic issue highlights the complex but important interactions of different cellular and molecular networks which governed the different key steps associated with inflammation-mediated cancer progression and metastasis through modulating the TIME. All these published articles discussed the essential role of inflammation-associated genes such as MMPs, CD200, and BRCA1/2 and their impact on myeloid biology in the context of TME. Altogether, these studies should lead to the development of novel molecular biomarkers and promising therapeutic targets for a more effective prognosis and therapeutic response to metastatic cancers. These studies are very innovative because they challenge current research paradigms and explore new immunotherapy-based intervention strategies for the treatment of metastatic cancers. However, we believe that studies focusing on the role of inflammation and myeloid cells that affects pre-metastatic niche formation, cancer cells dissemination, and immune evasion are lacking in this Research Topic. Understanding whether inflammation-mediated regulation of myeloid cells affects immune evasion and pre-metastatic niche formation in different human malignancies should be investigated and discussed in these articles.
